# Femoral nerve block versus fascia iliaca block for pain control in knee and hip arthroplasties: A meta-analysis

**DOI:** 10.1097/MD.0000000000025450

**Published:** 2021-04-09

**Authors:** Xiaohua Fan, Fei Cao, Ailin Luo

**Affiliations:** a Department of Anesthesiology, Tongji Hospital of Tongji Medical College, Huazhong University of Science and Technology; b Department of Orthopaedics, Chengdu First People's Hospital, China.

**Keywords:** fascia iliaca block, femoral nerve block, meta-analysis, total joint arthroplasty

## Abstract

**Background:**

This meta-analysis aimed to compare the efficiency of fascia iliaca compartment block (FICB) and femoral nerve block (FNB) for pain management in knee and hip surgeries.

**Methods:**

We searched four electronic databases (Pubmed, Embase, Cochrane library database, Web of Science) from inception to January 2019. Only randomized controlled trials (RCTs) were included. Two review authors independently extracted data for each included study. Primary outcomes were visual analogue scale at 12 hours, 24 hours, 48 hours, total morphine consumption, the length of hospital stay and the occurrence of nausea and vomiting. Standardized mean difference (SMD) or risk ratio (RR) and 95% confidence intervals (CIs) were calculated for continuous outcomes and discontinuous outcomes respectively. We used the Cochrane Risk of Bias tool to assess risk of bias. Stata 12.0 was used for meta-analysis.

**Results:**

Finally, 7 RCTs involving 508 patients (FICB = 254, FNB = 254) were included in this meta-analysis. Compared with FNB group, FICB has no benefit for visual analogue scale at 12 hours (SMD = 0.02, 95% CI, −0.15 to 0.19; *P* = .820), 24 hours (SMD = −0.02, 95% CI, −0.22 to 0.18; *P* = .806), and 48 hours (SMD = −0.02, 95% CI, −0.22 to 0.19; *P* = .872). No significant differences were found regarding total morphine consumption (SMD = −0.07, 95% CI, −0.29 to 0.15; *P* = .533). What's more, there was no significant difference between the length of hospital stay and the occurrence of nausea and vomiting (*P* > .05).

**Conclusion:**

FICB has equivalent pain control and morphine-sparing efficacy when compared with FNB. More high-quality RCTs are needed to identify the optimal drugs and volume of local infiltration protocols.

## Abstract 2

Supplemental Digital Content is available in the text

## 1 Introduction

Total knee arthroplasty (TKA) and total hip arthroplasty (THA) are common surgical procedures for the treatment of the degenerative disorders and traumatic diseases.^[1]^ Despite the obvious benefits of TKA and THA, there are still many intractable problems such as pain and vomiting after operation.^[2]^ Usually, several pain management protocols are used to relieve postoperative pain, such as local infiltration anesthesia,^[3]^ peripheral nerve blocks,^[4]^ epidural anesthesia,^[5]^ and multimodal analgesia.^[6,7]^ However, there is still no uniform gold standard for effective pain management after total knee and hip arthroplasty. Therefore, postoperative pain management after total knee and hip arthroplasty is still a controversial topic in the field of the joint procedure.

Femoral nerve block (FNB) and fascia iliaca compartment block (FICB) were 2 common pain management in TKA and THA. FNB has a potential for injury to the femoral vessels. And studies shown that FICB avoids these complications by anesthetizing the femoral nerve remotely from important neurovascular structures while still providing adequate analgesia.^[8]^ Whether FICB would be equivalent to FNB for analgesia in total knee and hip arthroplasty remains unclear due to a lack of published studies and small sample sizes. Therefore, we performed the present meta-analysis from randomized controlled trials (RCTs) to compare the efficiency and safety between FNB and FICB for postoperative pain control in total knee and hip arthroplasty.

The purpose of this meta-analysis was to perform a meta-analysis to identity whether FICB has an equivalent pain control efficacy and with less adverse effects than FNB.

## 2 Methods

The recommended preferred reporting items for systematic reviews and meta-analyses (PRISMA) statement were followed for the present systematic review and meta-analysis. Ethical approval was not necessarily needed as this is a meta-analysis based on previous studies.

### 2.1 Search strategies

Electronic literature searches, both manual and computer-assisted, were conducted using Pubmed, Embase, Cochrane library database, Web of Science from their date of inception to January 2019. Search terms used were: “femoral nerve block”, “FNB”, “femoral nerve catheters”, “total knee arthroplasty”, “TKA”, “total hip arthroplasty”, “THA”, “randomized controlled trials”, “controlled clinical trial”, “randomized”, “controlled trial”, “random”. The literature search was refined to randomized controlled trials (RCTs) in total knee and hip arthroplasty. Reference lists in studies, reviews, and previous meta-analyses were checked to identify any initially omitted studies. Two investigators independently reviewed the title, abstract, and full text of all articles.

### 2.2 Study eligibility criteria and exclusion criteria

Studies were included if they met the following criteria:

1. RCTs;
2. patients underwent total knee and hip arthroplasty;
3. FICB was compared with FNB;
4. studies have evaluated the efficacy or safety of FICB versus FNB using at least one of the following endpoints:
  a. Visual analogue scale (VAS) at 12 hours, 24 hours, 48 hours, total morphine consumption, the length of hospital stay and the occurrence of nausea and vomiting.

The following criteria were used for data exclusion:

1. retrospectively designed trials or trials of low quality;
2. letters, case reports, comments, meta-analysis, review and meeting abstracts;
3. data were unavailable to risk ratios (RR) or standardized mean difference (SMD)

### 2.3 Assessment of risk of bias

According to the Cochrane Handbook for Systematic Reviews of Interventions,^[9]^ two independent reviewers assessed methodological quality, and the following criterions were evaluated and given a grade of low, medium, or high risk bias: random sequence generation, allocation concealment, blinding of participants and personnel blinding of outcome assessment, incomplete outcome data, selective reporting and other bias. Based on the report and appropriateness of methods, the included studies were graded accordingly:

1. low risk (methods were indicated and proper),
2. high risk (methods were indicated but improper), and
3. moderate risk (methods were not indicated).

Disagreements on the risk of bias ratings were regularly resolved through discussion by the two reviewers.

### 2.4 Data extraction

Two investigators independently performed the data extraction. The data extracted included both study characteristics and measuring outcomes from the included studies. Study characteristics included the first author's name, year of publication, sample size, intervention, dose, transfusion indication, and surgical procedure. VAS at 12 hours, 24 hours, 48 hours, total morphine consumption, the length of hospital stay and the occurrence of nausea and vomiting were recorded as the measuring outcomes of the effectiveness of these two interventions. When data were incomplete or unclear, attempts were made to contact the investigators for clarification.

### 2.5 Statistical analysis

The current meta-analysis was calculated using Stata 12.0 (Stata Corp., College Station, TX). For continuous outcomes, the standard mean difference (SMD) with 95% confidence intervals (CIs) was used. For discontinuous outcomes, relative risk (RR) with 95% CIs was used. The statistical heterogeneity was assessed by the value of P and I^2^ using the chi-square test. If the I^2^ > 50% or *P* < .05 were considered to demonstrate significant heterogeneity and the random-effect model was chosen, otherwise the fixed-effect model was chosen. This meta-analysis also used a funnel plot, Begg test and Egger test of VAS at 12 hours to independently assess publication bias. Sensitivity analysis was also performed by omitting each included study in turn.

## 3 Results

### 3.1 Search results

**Figure 1:**
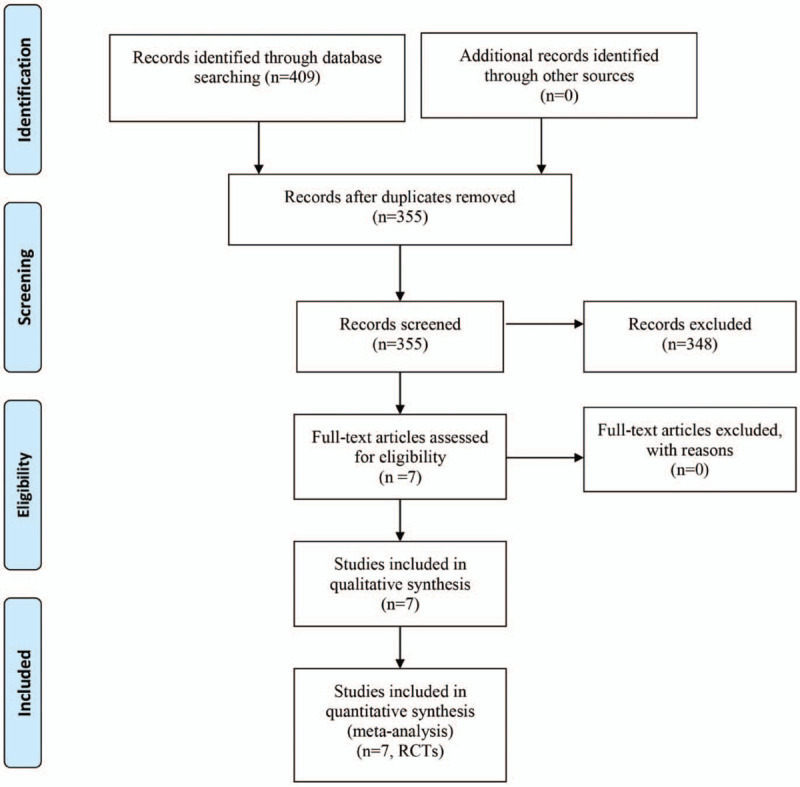
Flow of trials through the meta-analysis.

A total of 409 studies were identified through the initial search, and 54 papers were excluded due to the duplicates. In the next stage, 348 papers were excluded after reading the title and abstract. Therefore, 7 RCTs involving 508 patients (FICB = 254, FNB = 254, Fig. 1)^[10–16]^ were included after reading the full text according to the inclusion criteria.

### 3.2 Study characteristics

The baseline characteristics of the included studies are summarized in Table 1. All the studies were published between 2010 and 2018. The sample size ranged from 62 to 80. Six studies administration general anesthesia and one study did not report which anesthesia was adopted. The volume of drugs ranged from 20 ml to 60 ml. Most of the studies use ropivacaine for pain control. Three studies perform THA surgery, 3 studies perform TKA surgery and the remain one perform fractured neck of femur. All of the included studies were RCTs. Follow-up duration ranged from 1 month to 4 months.

**Table 1:** General characteristic of the included studies.

Author	Country	No. of patients (n)	Mean age (yr)	Anesthesia	Anesthesia protocol of FNB	Anesthesia protocol of FIB	Patients	Study type	Outcomes	Follow-up
Cooper, 2018	Australia	52/48	80/84	NS	NS	NS	fractured neck of femur	RCTs	1,2,3,4,5,6	NS
Kanadli, 2018	Turkey	50/50	75/73.4	General anesthesia	60 mL of 0.2% ropivacaine	60 mL of 0.2% ropivacaine	TKA	RCTs	1,2,3,5,6	3 months
Lončar-Stojiljković, 2016	Switzerland	15/15	70.4/71.2	General anesthesia	40 mL 0.75% ropivacaine	40 mL 0.75% ropivacaine	THA	RCTs	1,5,6	3 months
McMeniman, 2010	Australia	47/51	67.7/68.2	General anesthesia	60 mL of 0.2% ropivacaine	60 mL of 0.2% ropivacaine	TKA	RCTs	2,3,5,6	3 months
Möller, 2013	Australia	40/40	64/62	General anesthesia	50 mL prilocaine	50 mL prilocaine	TKA	RCTs	1,4,5,6	3 months
Yu, 2016	China	30/30	79.9/80.6	General anesthesia	20 mL 0.5% ropivacaine	20 mL 0.5% ropivacaine	THA	RCTs	2,3,4,6	1 months
Deniz, 2014	Turkey	20/20	67.8/59.1	NS	2% prilocaine, 30 ml of 0.25% bupivacaine	2% prilocaine, 30 mL of 0.25% bupivacaine	THA	RCTs	1,2,4,5,6	4 months

1, VAS at 12 hours, 2 VAS at 24 hours, 3, VAS at 48 hours, 4 total morphine consumption, 5, the length of hospital stay, 6, the occurrence of nausea. NS = not stated, THA = total hip arthroplasty, TKA = total knee arthroplasty.

### 3.3 Risk of bias in included studies

**Figure 2:**
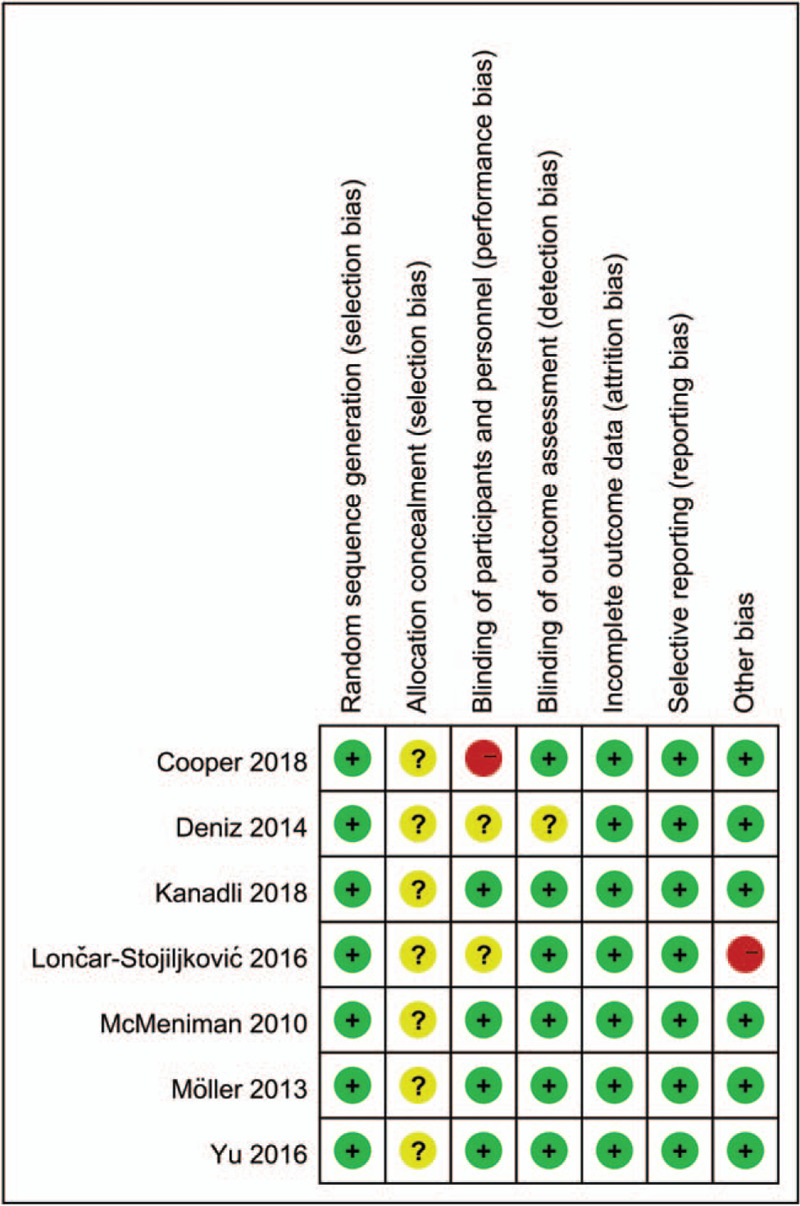
A, the risk of bias summary, +, no bias; –, bias;?, bias unknown.

**Figure 3:**
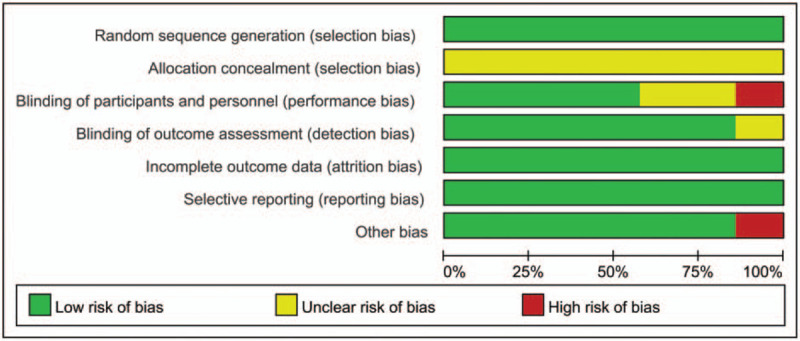
Risk of bias of summary of the included randomized controlled trials.

Figures 2 and 3 summarize the risk of bias summary and risk of bias graph of the 7 included studies. All the RCTs described the random sequence generation and listed as low risk of bias. Risk of bias for allocation concealment was unclear in all of the included studies. Blinding of the participant was with high risk of bias in one study. Blinding of the participant was unclear in one study. One study included a sample less than 15 and thus identify as high risk of bias.

## 4 Meta-analysis results

### 4.1 VAS scores at 12 hours

**Figure 4:**
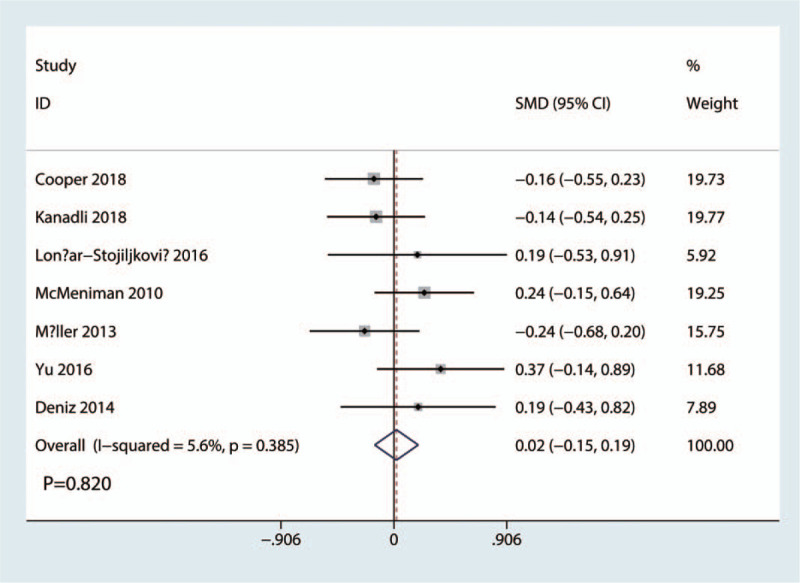
Forest plots of the included studies comparing the VAS scores at 12 hours.

VAS scores at 12 hours was reported in 7 studies with a total of 508 patients: 254 patients in the FICB group and 254 patients in the FNB group. The results of the meta-analysis showed there was no significant difference between FICB and FNB groups in terms of the VAS at 12 hours (SMD = 0.02, 95% CI, −0.15 to 0.19; *P* = .820, Fig. 4). A fixed-effects model was applied due to the low statistical heterogeneity in the meta-analysis (*P* = .385, I^2^ = 5.6%).

### 4.2 VAS scores at 24 hours

**Figure 5:**
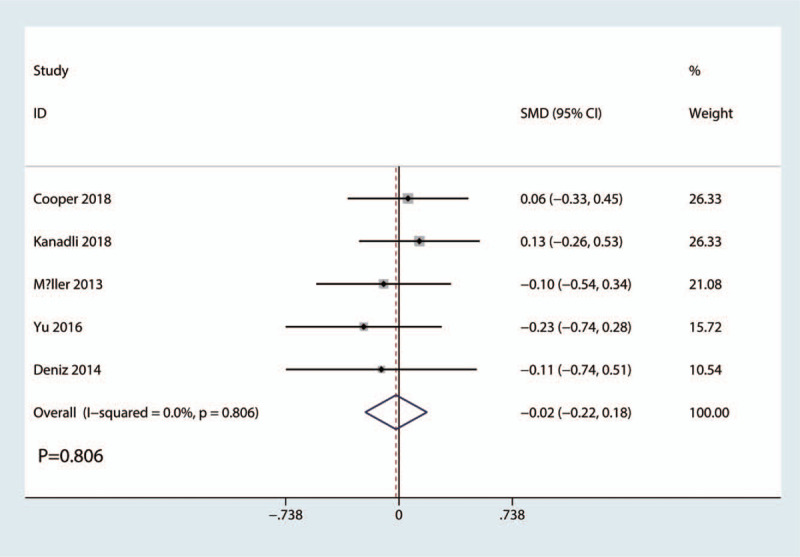
Forest plots of the included studies comparing the VAS scores at 24 hours.

VAS scores at 24 hours was reported in 5 studies with a total of 380 patients: 192 patients in the FICB group and 188 patients in the FNB group. The results of the meta-analysis showed there was no significant difference between FICB and FNB groups in terms of the VAS at 24 hours (SMD = −0.02, 95% CI, −0.22 to 0.18; *P* = .806, Fig. 5). A fixed-effects model was applied due to the low statistical heterogeneity in the meta-analysis (*P* = .806, I^2^ = 0.0%).

### 4.3 VAS scores at 48 hours

**Figure 6:**
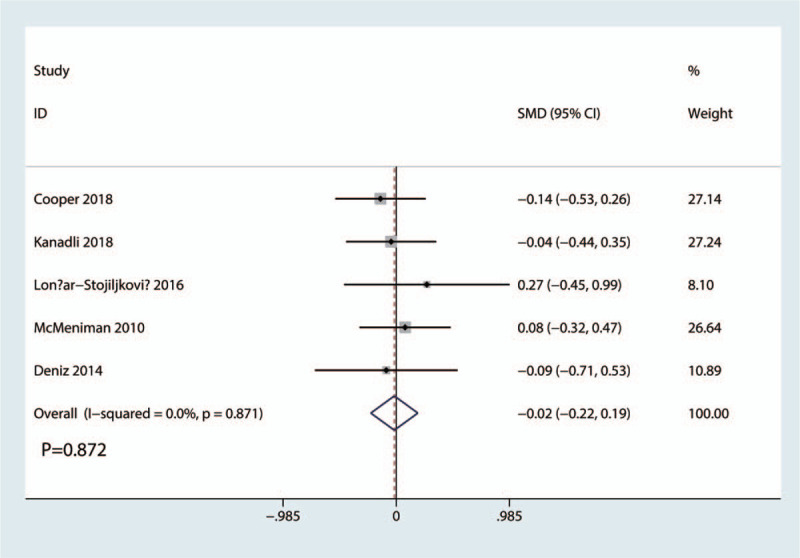
Forest plots of the included studies comparing the VAS scores at 48 hours.

VAS scores at 48 hours was reported in 5 studies with a total of 368 patients: 184 patients in the FICB group and 184 patients in the FNB group. The results of the meta-analysis showed there was no significant difference between FICB and FNB groups in terms of the VAS at 48 hours (SMD = −0.02, 95% CI, −0.22 to 0.19; *P* = .872, Fig. 6). A fixed-effects model was applied due to the low statistical heterogeneity in the meta-analysis (*P* = .871, I^2^ = 0.0%).

### 4.4 Total morphine consumption

**Figure 7:**
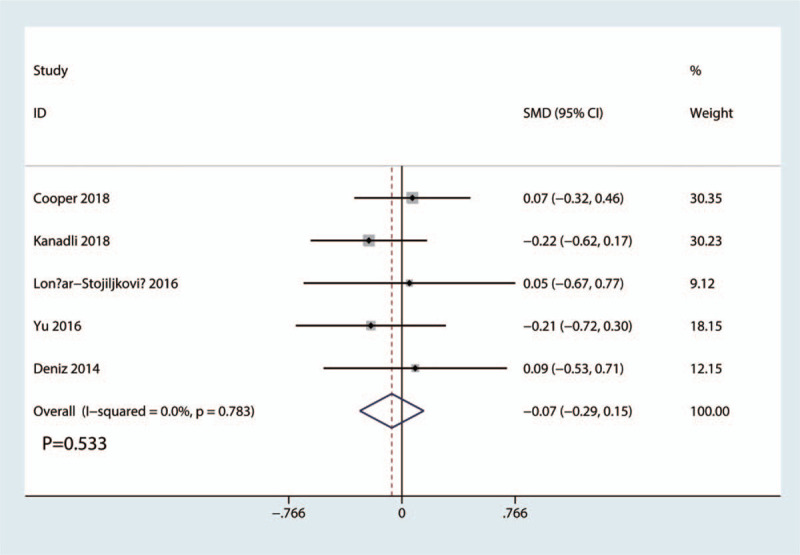
Forest plots of the included studies comparing the total morphine consumption.

Total morphine consumption was reported in 5 studies with a total of 330 patients: 167 patients in the FICB group and 163 patients in the FNB group. The results of the meta-analysis showed there was no significant difference between FICB and FNB groups in terms of the total morphine consumption (SMD = −0.07, 95% CI, −0.29 to 0.15; *P* = .533, Fig. 7). A fixed-effects model was applied due to the low statistical heterogeneity in the meta-analysis (*P* = .783, I^2^ = 0.0%).

### 4.5 Length of hospital stay

**Figure 8:**
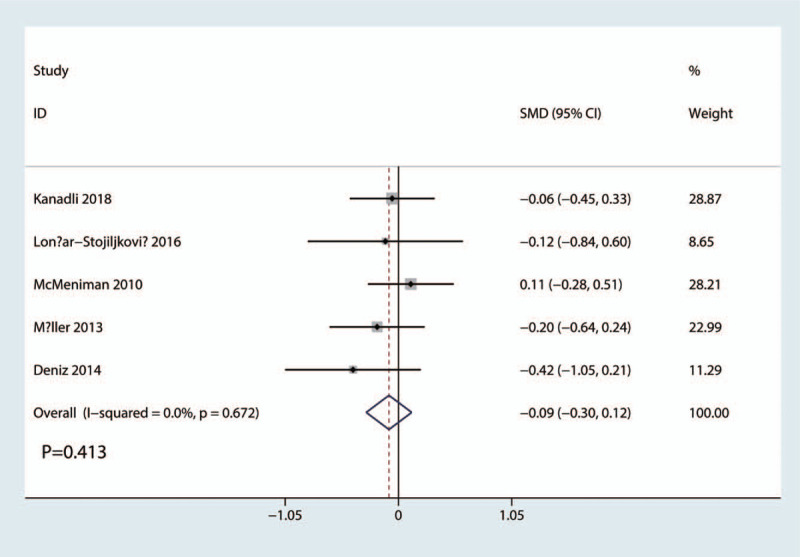
Forest plots of the included studies comparing the length of hospital stay.

Length of hospital stay was reported in 5 studies. The results of the meta-analysis showed there was no significant difference between FICB and FNB groups in terms of the length of hospital stay (SMD = −0.09, 95% CI, −0.30 to 0.12; *P* = .413, Fig. 8). A fixed-effects model was applied due to the low statistical heterogeneity in the meta-analysis (*P* = .672, I^2^ = 0.0%).

### 4.6 Nausea and vomiting

**Figure 9:**
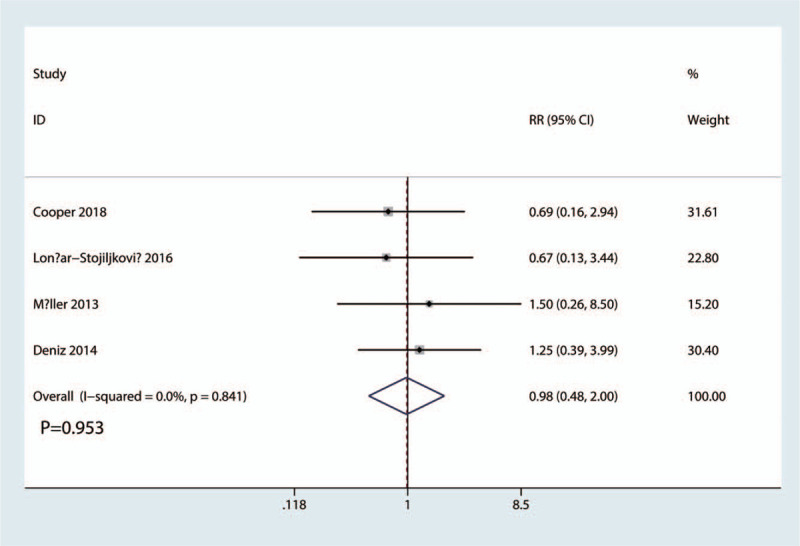
Forest plots of the included studies comparing the occurrence of nausea and vomiting.

Occurrence of nausea and vomiting was reported in 4 studies. The results of the meta-analysis showed there was no significant difference between FICB and FNB groups in terms of the occurrence of nausea (RR = 0.98, 95% CI, 0.48 to 2.00; *P* = .953, Fig. 9). A fixed-effects model was applied due to the low statistical heterogeneity in the meta-analysis (*P* = .841, I^2^ = 0.0%).

### 4.7 Publication bias, Sensitivity analysis, and subgroup analysis

**Figure 10:**
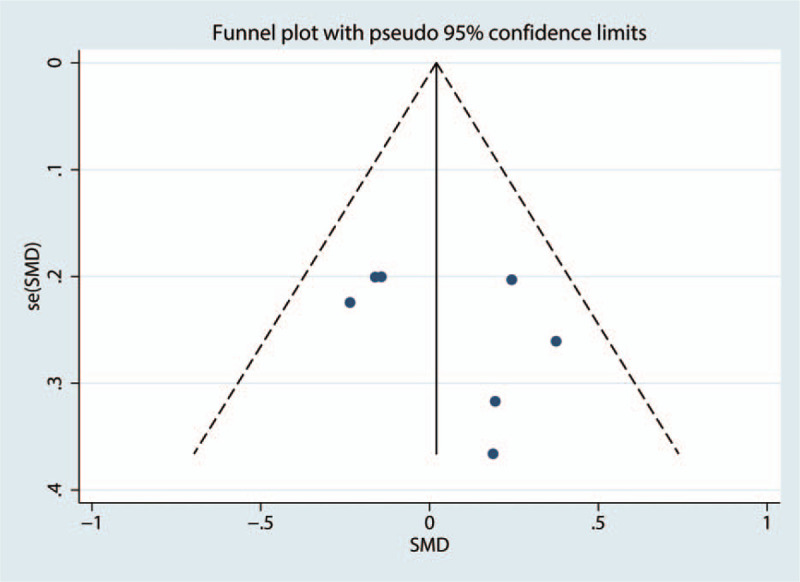
Funnel plot of the VAS score at 12 hours.

**Figure 11:**
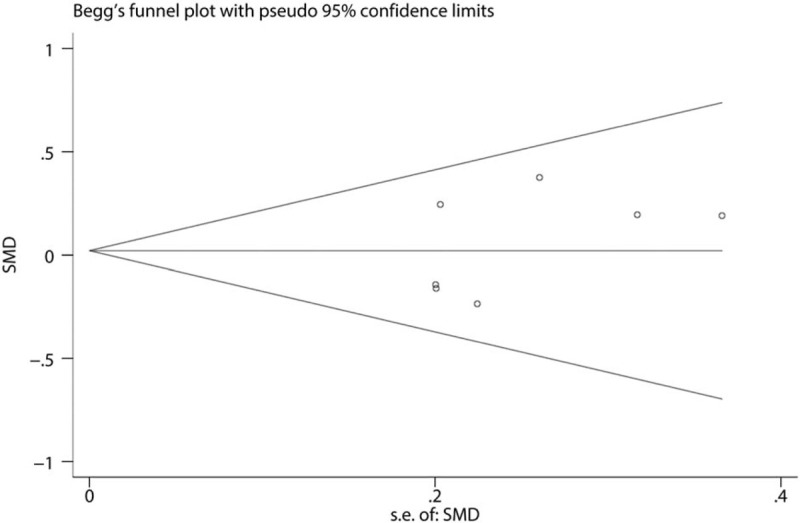
Begg test of the VAS score at 12 hours.

**Figure 12:**
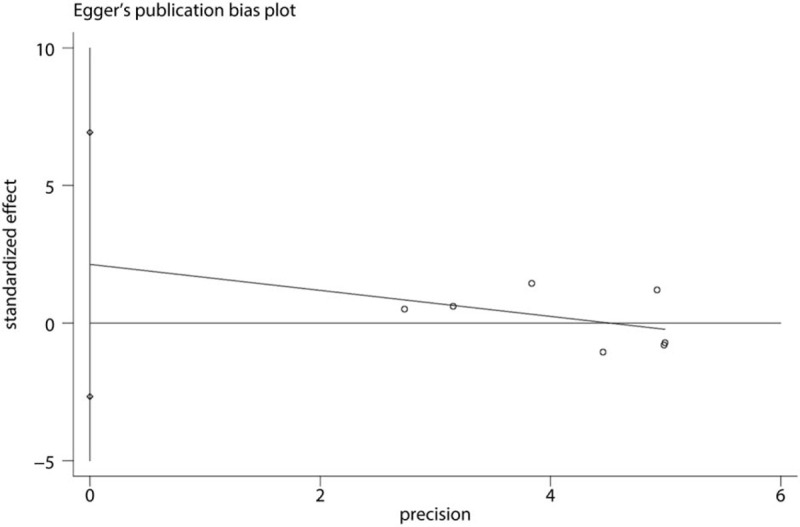
Egger test of the VAS score at 12 hours.

Funnel plot of the VAS at 12 hours was shown in Figure 10. Results shown that effect size was symmetrical and thus no publication bias was existed in this meta-analysis. We further used Begg test (Fig. 11) and Egger test (Fig. 12), result found that there was no publication bias.

**Figure 13:**
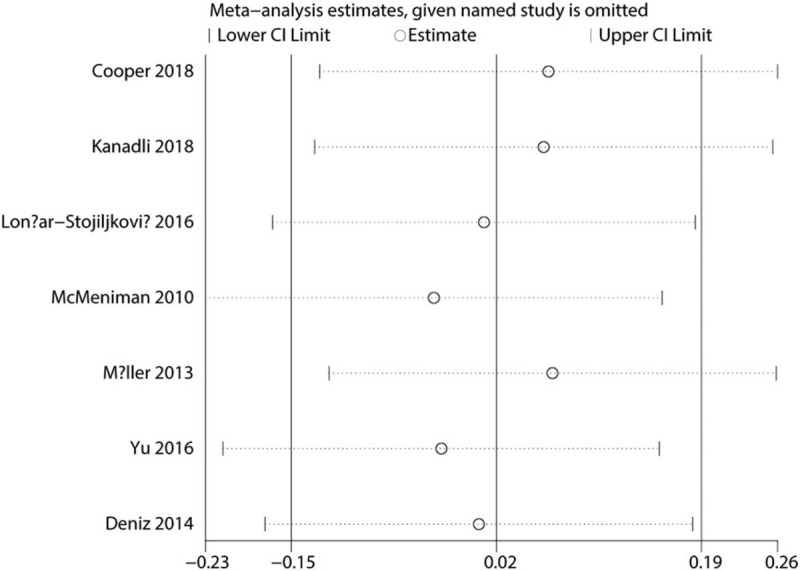
Sensitivity analysis of the VAS score at 12 hours.

Sensitivity analysis was performed by omitting each study in turn and results found that after omitting each study in turn, overall effects was not changed (Fig. 13).

Subgroup analysis results found that there was no subgroup difference between different subgroups (anesthesia method (see Supplementary Digital Content, 1, http://links.lww.com/MD2/A39, subgroup analysis for anesthesia methods for VAS at 12 hours), volume of local infiltration (see Supplementary Digital Content, 2, http://links.lww.com/MD2/A40, subgroup analysis for volume of local infiltration for VAS at 12 hours) and surgery type (see Supplementary Digital Content, 3, http://links.lww.com/MD2/A41, subgroup analysis for surgery type for VAS at 12 hours)).

## 5 Discussion

This meta-analysis revealed that FICB and FNB achieved a clinically significant mean pain score reduction, with no apparent difference between the two. Moreover, no statistically significant between the two in terms of the total morphine consumption and occurrence of nausea and vomiting. Sensitivity analysis and subgroup analysis further confirmed these conclusions.

FNB has previously demonstrated adequate perioperative pain relief as well as rapid rehabilitation in the postoperative period.^[17–19]^ And studies have shown that FNB was superior than patient-controlled anesthesia for TKA patients.^[20]^ However, FNB has a risk of severe neuraxial compression. In recent years, FICB was introduced and shown that has a positive role in decreasing pain intensity after hip fracture, TKA and THA patients. Whether FICB was superior to FNB in reducing pain intensity was unknown.

Wang et al^[21]^ conducted a meta-analysis about FICB and FNB in hip and knee arthroplasties. However, they only included 5 RCTs for meta-analysis. Hong et al^[22]^ suggested that FICB has a beneficial role in reducing pain intensity and morphine consumption after hip fracture than the control group. Current meta-analysis has shown that FICB has an equivalent pain control in hip and knee surgeries. We compared VAS scores at 12 hours, 24 hours, and 48 hours. Results found that no significant difference was found at 12 hours, 24 hours, and 48 hours after surgery. Temelkovska-Stevanovska et al^[23]^ identified that pain relief in the postoperative period was superior in the FNB group versus the FICB group at rest and in movement in patients with hip fracture. Wang et al^[21]^ found that FICB has similar results with our meta-analysis. When FICB was compared with a 3-in-1 block for anterior cruciate ligament reconstruction, Morau et al^[24]^ demonstrated no significant difference in postoperative VAS pain scores between FICB and control.

We then test the total morphine consumption between FICB and FNB groups. Results have shown that FICB and FNB have no statistically significance between the two groups. We only calculated total morphine consumption in hospital. Temelkovska-Stevanovska et al^[23]^ revealed that there was no significant difference between FICB and FNB groups in terms of the total morphine consumption.

We also included the length of hospital stay as an outcome. Since effective pain relief could decrease the length of hospital stay. Results have shown that there was no significant difference between the length of hospital stay between FICB and FNB groups. No significant difference was found regarding postoperative nausea and vomiting. Due to the small number of included articles, large sample sizes of high-quality RCTs are further needed.

The present meta-analysis exists some limitations that should be noted.

1. Only 7 RCTs were included in the present meta-analysis, although all of them are recently published RCTs, the sample size are relatively small (n < 100);
2. Dose and volume of local anesthetics are varied, which may influence the results;
3. the duration of follow up is relatively short which leads to underestimating complications;
4. Though funnel plot and Begg's test found that there was no publication bias in present meta-analysis, due to the limited number of the included studies, publication bias needs to be further identified.

## 6 Conclusion

FICB provides equal postoperative pain control compared with FNB following knee and hip surgeries. Both of them can reduce the consumption of opioids without severe adverse effects. More high-quality large RCTs with long follow-up period are necessary for proper comparisons of the efficacy and safety of FNB with FICB.

## Author contributions

XHF and FC conceived the study design. ALL and XHF performed the study, collected the data and contributed to the study design. XHF and FC prepared the manuscript. All edited the manuscript.

All authors read and approved the final manuscript.

**Resources:** Xiaohua Fan.

**Software:** Xiaohua Fan, Fei Cao.

**Supervision:** Fei Cao, Ailin Luo.

**Validation:** Ailin Luo.

**Visualization:** Ailin Luo.

**Writing – original draft:** Ailin Luo.
